# Unique wing scale photonics of male Rajah Brooke’s birdwing butterflies

**DOI:** 10.1186/s12983-016-0168-7

**Published:** 2016-08-12

**Authors:** Bodo D. Wilts, Marco A. Giraldo, Doekele G. Stavenga

**Affiliations:** 1 Adolphe Merkle Institute, University of Fribourg, Chemin des Verdiers 4, CH-1700 Fribourg, Switzerland; 2 Biophysics Group, Institute of Physics, University of Antioquia, Calle 70 No. 52-21, A.A.1226, Medellín, Colombia; 3 Computational Physics, Zernike Institute for Advanced Materials, University of Groningen, Nijenborgh 4, NL-9747AG Groningen, The Netherlands

**Keywords:** Animal coloration, Polarization, Grating, Iridescence, Diffraction, FDTD, Photonics, Melanin

## Abstract

**Background:**

Ultrastructures in butterfly wing scales can take many shapes, resulting in the often striking coloration of many butterflies due to interference of light. The plethora of coloration mechanisms is dazzling, but often only single mechanisms are described for specific animals.

**Results:**

We have here investigated the male Rajah Brooke’s birdwing, *Trogonoptera brookiana*, a large butterfly from Malaysia, which is marked by striking, colorful wing patterns. The dorsal side is decorated with large, iridescent green patterning, while the ventral side of the wings is primarily brown-black with small white, blue and green patches on the hindwings. Dense arrays of red hairs, creating a distinct collar as well as contrasting areas ventrally around the thorax, enhance the butterfly’s beauty. The remarkable coloration is realized by a diverse number of intricate and complicated nanostructures in the hairs as well as the wing scales. The red collar hairs contain a broad-band absorbing pigment as well as UV-reflecting multilayers resembling the photonic structures of *Morpho* butterflies; the white wing patches consist of scales with prominent thin film reflectors; the blue patches have scales with ridge multilayers and these scales also have centrally concentrated melanin. The green wing areas consist of strongly curved scales, which possess a uniquely arranged photonic structure consisting of multilayers and melanin baffles that produces highly directional reflections.

**Conclusion:**

Rajah Brooke’s birdwing employs a variety of structural and pigmentary coloration mechanisms to achieve its stunning optical appearance. The intriguing usage of order and disorder in related photonic structures in the butterfly wing scales may inspire novel optical materials as well as investigations into the development of these nanostructures in vivo.

## Background

Butterflies are a hallmark of biodiversity and multifunctionality in nature. Of particular beauty are the birdwing butterflies of Australasia, which are noted by their exceptional size and a birdlike flight [1, 2]. An especially attractive butterfly is Rajah Brooke’s birdwing, *Trogonoptera brookiana*, the national butterfly of Malaysia. However, a detailed explanation of the striking coloration has yet to be made.

Recent studies on butterfly coloration have revealed a multitude of optical mechanisms that strongly alter the composition of incident light and reflect strong colors. Pigments are generally the major means to create color. The pigments encountered in butterflies are rather family specific. For instance, pterins are prominently encountered in the Pieridae [3], ommochromes are the pigments of the Nymphalidae [4], while papiliochrome pigments have been only found in Papilionidae [5–8]. The pigments are dispersed in scales that cover butterfly wings like tiles on a roof. A butterfly wing scale basically consists of a lower lamina, which is essentially a thin plate with thickness 100-200 nm, connected by pillar-like trabeculae to the upper lamina, which is made up of an array of parallel ridges, with interdistance 1-2 μm, and connecting cross-ribs [9].

The fine structure of the scales often creates structural coloration. For instance, the lower lamina of the scales universally acts as a thin film reflector [10, 11]. Furthermore, the ridges of the upper lamina are built of lamellae, which in most *Morpho* species have extensive overlap [12, 13]. The stacked lamellae then act as optical multilayers, which create the butterflies’ striking blue-metallic reflections. Multilayers with perforations exist in the scale lumen of many lycaenids [14, 15]. Intricate structures, acting as three-dimensional photonic crystals, have been demonstrated in several lycaenid as well as papilionid species [16–19].

We recently reported the coloration mechanisms of the *Ornithoptera*, a genus of birdwings closely related to *Trogonoptera sp.* [1], with colorful scales consisting of a large membrane stack that acts as a chirped multilayer [7], where the reflected light is filtered by papiliochrome pigments. A previous study also reported the intriguingly complex ultrastructure of the strongly green reflecting wing scales of the birdwing butterfly *T. brookiana* [20]. The anatomy of the birdwing scales has inspired the production of advanced materials via replication into inorganic materials [21–24]. However, we found that the latter papers contain confusing data and erroneous identifications of the scales of *T. brookiana*. Here we specifically focus at the optics of *T. brookiana*’s green reflecting scales, but we also analyze the interplay of pigmentary and structural coloration realized in other wing scale types, using spectrophotometry, imaging scatterometry, and transmission and scanning electron microscopy. We demonstrate that the lower lamina of the white and black scales acts as a thin film blue reflector and that the ridges of the blue scales and red hairs function as multilayer reflectors. The uniquely structured green-iridescent scales contain complex three-dimensional photonic crystals, the optical signature of which could be understood by applying finite-difference time-domain modelling.

## Results

### Overall appearance of *Trogonoptera brookiana*

**Fig. 1:**
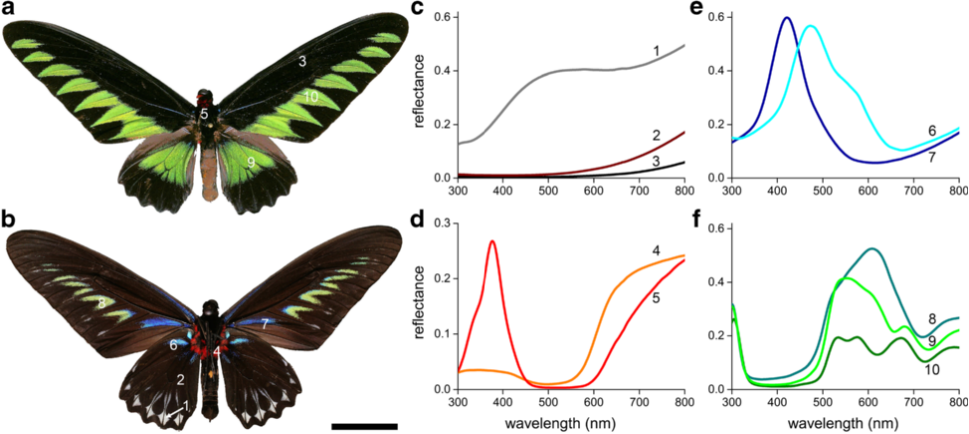
Rajah Brooke’s birdwing, *Trogonoptera brookiana*, and reflectance spectra. **a**, **b** Photographs of the upper (*dorsal*) and lower (*ventral*) side of a male. **c**-**f** Reflectance spectra; the number of the spectra corresponds to the wing and body location indicated in (**a**, **b**). Scale bar: **a**, **b** 5 cm

The upperside of the wings of the male Rajah Brooke’s birdwing, *T. brookiana*, is marked by mainly jet-black margins, which on the dorsal forewings surround a row of seven tooth-shaped green areas and on the hindwings border a large green patch. A bright red collar exists in the border area between head and thorax (Fig. 1a).

The underside of the wings is mainly dark-brown, with a few radiating, vivid-blue lines. A row of yellow-green chevrons with blue borders is present in the middle of the forewings. The hindwings are mainly black with at the outer rim a row of white spots. Red patches surround the thorax (Fig. 1b).

We measured the reflectance spectra of the various colored areas with a bifurcated fiber-probe spectrophotometer, which yielded intriguing spectral differences, especially for the green patches (Fig. 1c-f). Below we describe the very different pigmentary and structural aspects that contribute to the different colors, and we explain the resulting optical effects.

### Thin films in the white and black scales

**Fig. 2:**
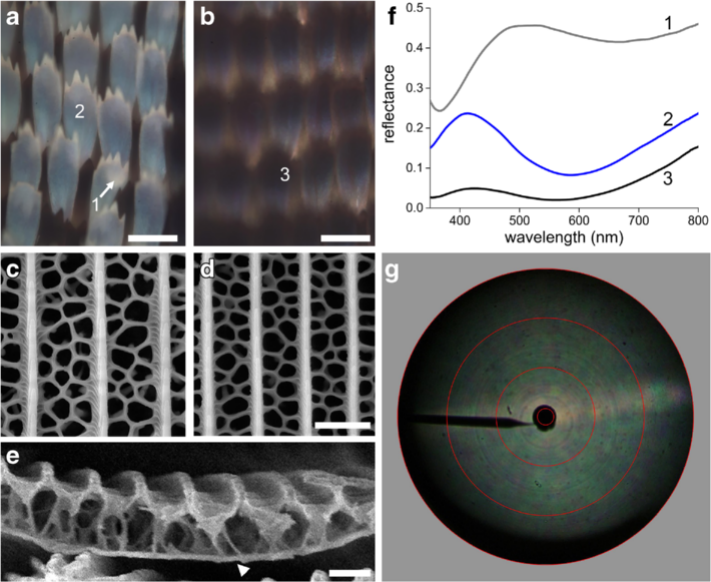
White and black scales. **a**, **b** Epi-illumination light micrographs of wing areas with white (area 2 of Fig. 1b) and black cover scales (area 3 of Fig. 1b). **c**, **d** Scanning electron micrographs of a white and a black scale, respectively. **e** Cross-sectional SEM image of a white scale showing the basic scale architecture with the lower lamina of thickness ~200 nm (arrowhead). **f** Reflectance spectra; spectrum 1 is from a white scale overlapping a white scale, and spectrum 2 is from a central area of a white scale above a black ground scale, as indicated in (**a**), while spectrum 3 is from a central area of a black scale above a black ground scale, as indicated in (**b**). **g** Scatterogram of a white scale. The red circles indicate directional angles of 5°, 30°, 60°, and 90°. Scale bars: **a**, **b** 100 μm, **c**-**e** 2 μm

Light microscopy of a white area (Fig. 1b, #1) shows that the color resides in a lattice of whitish cover scales overlapping ground scales that are either white or black (Fig. 2a, b). In the latter case the cover scales have a bluish hue. Interestingly, the scales of the brown-black areas (Fig. 1a, b, #2, 3; Fig. 2b) also display a bluish hue, although much weaker. Reflectance spectra measured with a microspectrophotometer revealed the pigmentary and/or structural origin of the different colors. The spectrum measured from a white cover scale overlapping a white ground scale showed a slightly undulating, broad-band spectrum (Fig. 2f, #1) similar as that measured with the bifurcated fiber probe (Fig. 1c, #1). However, a white cover scale overlapping a black ground scale yielded a very different, blue-peaking spectrum (Fig. 2f, #2), which resembles that of a chitinous thin film reflector with thickness ~200 nm [10, 25]. As demonstrated for several butterfly wing scales, the candidate thin film reflector is undoubtedly the scale’s lower lamina [10, 11].

To ascertain the structure of the scales of the whitish wing areas of *T. brookiana*, we performed scanning electron microscopy. The white cover and ground scales as well as the black ground scales showed the common papilionid wing scale structure, with a disordered cross-rib lattice connecting regularly spaced ridges (Fig. 2c-e; see further [5, 26, 27]).

The reflectance spectra measured of black scales (Fig. 2f, #3) resembled that of a white cover scale when on a black ground scale (Fig. 2f, #2) except for a much lower amplitude. The latter is evidently due to a high concentration of melanin in the scales’ upper lamina, which strongly reduces the light flux reaching the lower lamina as well as filters the reflected light flux. The lower lamina’s thickness of ~200 nm, suggested by the reflectance spectrum, was confirmed by scanning electron microscopy (Fig. 2e).

Thin film reflectors, like those encountered in soap bubbles, usually reflect incident light very directionally, but this is not the case for the scales when illuminated from the side of the upper lamina, because the disordered cross-rib lattice acts as a diffuser. This was directly demonstrated by imaging scatterometry, using a narrow aperture illumination, which yielded a broad, diffuse pattern with a faint horizontal line caused by light diffraction at the ridge grating (Fig. 2g).

### Pigmentary and structurally colored red hairs

**Fig. 3:**
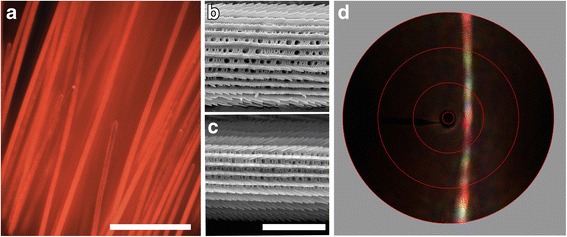
Red hairs. **a** Epi-illumination light micrograph of the red hairs near the abdomen (area 4 of Fig. 1b); scale bar 100 μm. **b**, **c** Scanning electron micrograph of red hairs of the area near the abdomen and collar, respectively; scale bar: 5 μm. **d** Scatterogram of a red hair

Light microscopic inspection of the red-colored ventral areas around the abdomen (Fig. 1b, #4) and the red collar area (Fig. 1a, #5) revealed very similar, densely-packed hairs (Fig. 3a). The reflectance spectra of both areas (Fig. 1d) showed a high reflectance at long wavelengths, consistent with papiliochrome pigment absorbing up to the red wavelength range [5, 26]; the spectral separation of spectra # 4 and #5 in Fig. 1d is most probably due to a higher amount of pigment in the hairs in the collar region. However, this explanation is at variance with the high UV-reflectance of the collar hairs (Fig. 1d, #5), which therefore suggests a structural origin.

To further investigate this, we performed scanning electron microscopy (Fig. 3b, c). As found in other cases, the ventral and collar hairs have a more or less cylindrical shape [9, 28]. The surface of the hairs featured prominent longitudinal ridges, very similar to the ridges of the white and black wing scales (e.g., Fig. 2c-e). However, in the cylindrical hairs the ridges are present along the complete circumference, while in the wing scales the ridges are only present in the upper lamina; the lower lamina is an approximately flat plate. As in the wing scales, the ridges of the hairs consist of overlapping lamellae. Notably however, the lamellae of the collar hairs overlap each other much more than those of the ventral hairs. This reminds us of the case of the yellow and orange wing scales of various pierid butterflies where overlapping ridge lamellae create UV-reflecting multilayers [29–32]. The UV-reflectance peak of the collar hairs has clearly the same structural basis.

Imaging scatterometry on wing scales with ridge multilayers demonstrated that the parallel ridges can act as a diffraction grating [32–34] (see also Fig. 2g). To investigate whether the hairs behave similarly, we performed scatterometry on single, isolated hairs (Fig. 3d). The scatterograms obtained from the two hair types both showed a colorful, line-shaped pattern perpendicular to the hair axis, clearly due to diffraction by the parallel ridges. The additional vague, diffuse red background is due to randomly scattered light filtered by the short-wavelength absorbing pigment in the connecting cross-ribs and additional underlying structures (cf. the scatterogram of *Hebomoia glaucippe;* Fig. 3c of ref. [32]).

### Multilayered ridges in the blue scales

**Fig. 4:**
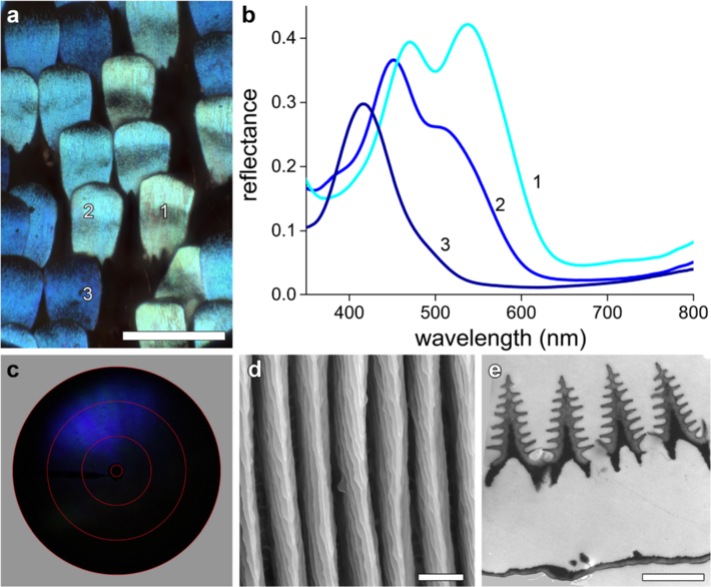
Optics and ultrastructure of the blue wing scales. **a** Epi-illumination light micrograph of an area with variously blue colored scales (area 6 in Fig. 1b). **b** Reflectance spectra of scales with numbers corresponding to those in panel (**a**). **c** Scatterogram (the red circles indicate scattering angles of 5, 30, 60, and 90°. **d** Scanning electron micrograph of a blue scale, showing high ridges. **e** Transmission electron micrograph of a blue scale, with an electron-dense core of the ridges. Scale bars: **a** 100 μm, **d**, **e** 1 μm

Epi-illumination light microscopy of the blue patches (Fig. 1b, #6, 7) showed that the scale lattice consists of blue-colored cover scales overlapping black ground scales (Fig. 4a). The color of the individual blue scales rather varies, which is of course reflected in the measured reflectance spectra (Fig. 4b). The narrow bands and fine structure of the spectra closely resemble the reflectance spectra of the blue wing scales of *Morpho* and pierid butterflies that have multilayered ridges [12, 31, 35].

As noted above, imaging scatterometry of wing scales with multilayered ridges generally yield characteristic, line-shaped scatterograms. However, scatterograms of the blue scales of *T. brookiana* had a rather different appearance. Illumination with a narrow aperture beam yielded a reflected light beam with a restricted spatial distribution (Fig. 4c), indicating a special scale structure that reflects directionally.

We performed electron microscopy to unravel the structural basis of the blue scales’ colors. Somewhat surprisingly, scanning electron microscopy showed that the scale ridges consist of elaborate stacks of lamellae, similar to the well-known *Morpho* ridge multilayers (Fig. 4d). Transmission electron microscopy confirmed this, but revealed a material organization of the ridges more complex than that of the *Morpho* scales. At the ridge interior a highly electron dense medium exists, suggesting local deposition of melanin (Fig. 4e). Light microscopic observations as well as microspectrophotometry on blue scales immersed in refractive index matching fluid indeed revealed a substantial amount of melanin.

The melanin deposition in the center of the ridges will act as an optical isolation mechanism, which thus explains the restricted spatial spread of reflected light in the scatterogram (Fig. 4c). Below we will recognize a similar and even much stronger isolation mechanism in the green scales.

### Complex photonics of the green scales

**Fig. 5:**
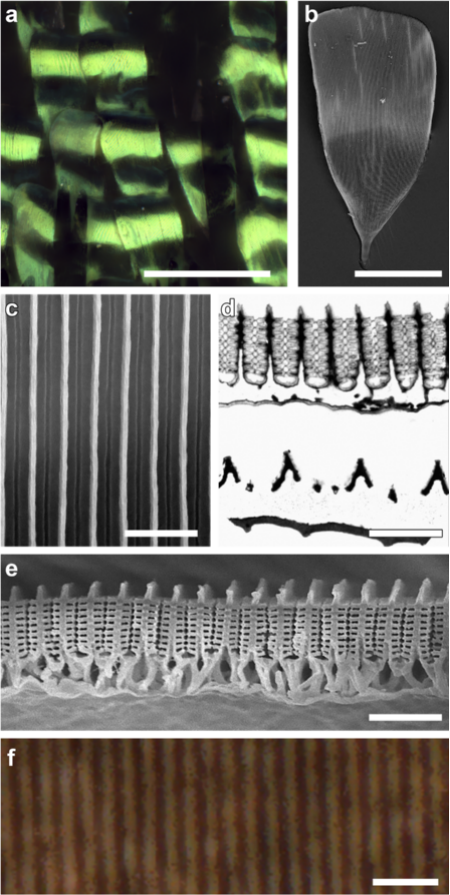
Structure of the green-colored wing scales. **a** Epi-illumination light micrograph of a green area (#10 of Fig. 1a). **b** Scanning elecron micrograph of a single green scale. **c** Close-up on-view SEM image showing ridges and minute gaps between them. **d** TEM cross-sectional image of a green cover scale and a black ground scale. **e** Cross-sectional SEM image of a green scale showing the extreme order of multiple ridges. **d** An isolated green wing scale immersed in refractive index matching fluid observed in transmitted light. Scale bars: **a** 100 μm. **b** 50 μm, **c**-**f**: 2 μm

The strongly variable reflectance spectra of the green patches (Fig. 1f, #8–10) betrayed complex structural properties of the wing scales. Epi-illumination light microscopy showed strongly curved scales with locally very bright reflections (Fig. 5a). Scanning electron microscopy of single scales demonstrated pronounced ridges, but the space in between the ridges was filled with an almost continuous layer, except for a narrow striped burrow (Fig. 5b, c).

Transmission electron microscopy revealed that the upper lamina of the green scales is indeed organized in a unique way. The lamellae fully fill the space in between neighboring ridges and even touch each other, so creating an almost continuous multilayer. This organization strongly deviates from that of ordinary papilionid scales, which normally have irregular arranged cross-ribs (e.g., Fig. 2c, d). Usually the laterally extending lamellae of the ridges are narrow and skewed with respect to the scale surface and have only limited overlap (compare the cross-sections of the green cover scale with the underlying black ground scale in Fig. 5d and the blue cover scale of Fig. 4d). That the elaborate ridges of the green scales have an extra-ordinary regular organization is also clear from a cross-section observed with scanning electron microscopy (Fig. 5e).

Interestingly, the ridge centers of the green scales contain a highly electron-dense material (Fig. 5d), like the blue scales (Fig. 4e). The suggested presence of melanin was demonstrated by embedding a scale in immersion oil and observing it with a light microscope using transmitted light (Fig. 5f). This revealed dark, brown-black stripes with distance of 0.8 μm, fully corresponding with the ridge distance (Fig. 5c-e). Absorbance measurements unequivocally confirmed the local concentration of melanin in the ridge centers.

**Fig. 6:**
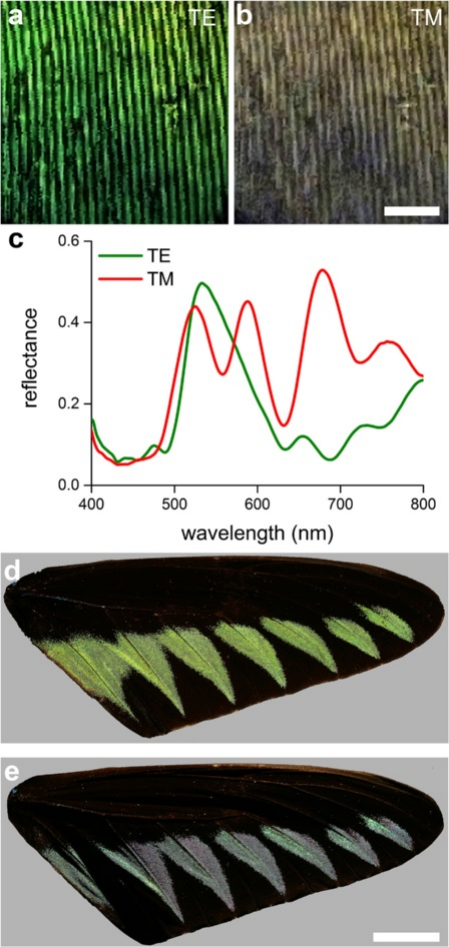
Polarizing optics of the green scales. **a**, **b** Local epi-illumination of a green scale with normally-incident light linearly-polarized perpendicular (TE) and parallel (TM) to the ridges. **c** Reflectance spectra corresponding to panel (**a**) and (**b**). **d**, **e** Photographs of a dorsal right wing under about perpendicular and oblique (~45°) illumination. Scale bars: **a**, **b** 5 μm, **d**, **e** 1 cm

The strongly anisotropic, longitudinal organization of the green scales suggested a strong polarization dependence of the scales’ optical properties. We investigated this by first applying from about a normal direction epi-illumination of a scale with TE- and TM-polarized light, i.e., light polarized perpendicularly and parallel to the ridges, respectively. That yielded distinctly different colors (Fig. 6a, b), as was documented also in the corresponding reflectance spectra measured with the microspectrophotometer (Fig. 6c). The high reflectivity of the wing scales and the polarization dependence suggested that the wing reflection characteristics could be strongly angle dependent. This could indeed be strikingly observed by illuminating a wing from different angular directions; huge shifts in hue can be elicited that way (Fig. 6d, e).

**Fig. 7:**
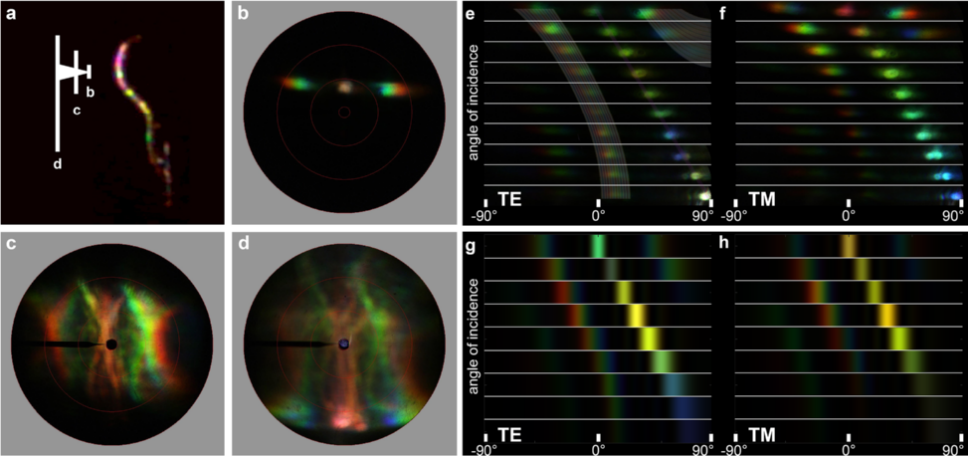
Imaging scatterometry of a green scale. **a** Side-view of a single scale glued to the end of a pulled glass micropipette. The width of the rectangles indicate the size of the illumination area used in panels **b**-**d. b**-**d** Scatterograms resulting from illumination of a small (**b**), medium-sized (**e**) and large (**f**) area. **e**, **f** Angle dependence of the diffraction patterns obtained with a narrow-aperture illumination beam (as in panel **b**) for TE- and TM-polarized light with the angle of light incidence increasing in ~7.5° steps. **g**, **h** Calculated angle dependence of the full photonic structure (see Fig. 8a, *iv*) for TE- and TM-polarization with the angle of light incidence varying from 0 to 80° in 10° steps

The distinct angle dependence of the scale reflectance might be fully due to the scale’s fine structure but could also be prominently affected by the scale’s strong curvature. To clarify this, we performed imaging scatterometry on a single, isolated green scale (Fig. 7). Local illumination on the central, flat part of the scale (diameter of illumination spot ~10 μm; Fig. 7a, arrow b) created a remarkably clear diffraction pattern (Fig. 7b). The diffraction pattern’s first order for 500 nm light was ~45°, which corresponds to a grating parameter of 0.77 μm, in excellent agreement with the measured ridge distance. Illumination with larger spots (diameter 30 and 60 μm; Fig. 7a, arrows c and d) yielded fuzzier scatterograms (Fig. 7c, d), although the principal diffraction pattern remained well-recognizable. Here, the shape of the scale severely affected the scattering pattern, as the strong curvature of the scale stretched the scattering pattern across the hemisphere (see also [32]).

We furthermore investigated the diffraction pattern as a function of the angle of illumination and the polarization. To allow this, we inserted a linear polarizer and a pinhole into the secondary illumination beam of the imaging scatterometer, allowing illumination with polarized light from angles of incidence between 0° and 90°. Figure 7e, f shows the angle-dependency for TE- and TM-polarized light, respectively. As expected, when the angle of incidence increases, the diffraction pattern also shifts to larger angles. More importantly, the color of the 0^th^ order reflection strongly varies with polarization and angle of incidence. Unexpectedly, the color change of the 0^th^ order reflection deviates from the typical iridescence of a multilayer, i.e., a blue shift with increasing angle of incidence). Somewhat erratically, the reflection first shifts to the red (with a maximum in-between 40 and 50°) before shifting to the blue. The angle-dependency of the diffraction pattern, i.e., the 0^th^, 1^st^ and -1^st^ diffraction order, can be well understood as to be from a grating with a ridge distance of ~0.77 μm (shaded bands overlaid in Fig. 7e).

### FDTD modelling of the photonic response

**Fig. 8:**
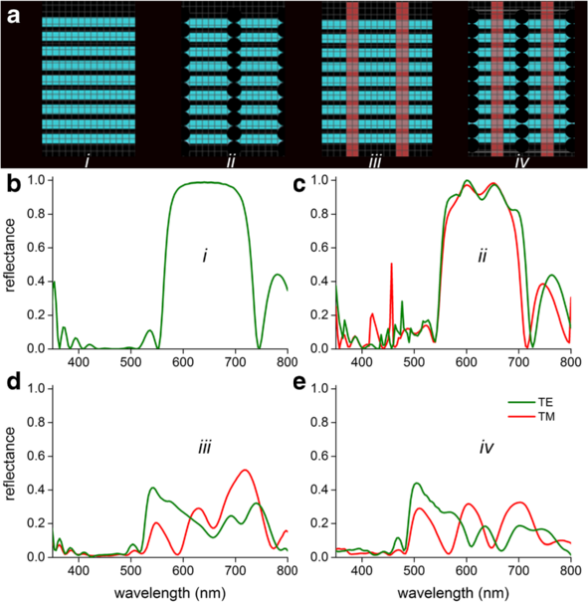
FDTD modelling of multilayered scale structures. **a** Sketches of four investigated topologies: (*i*) ideal multilayer of chitin (*cyan*) and air spaces, (*ii*) multilayers with air holes (*white*), (*iii*) multilayers with orthognal melanin baffles (*brown*), and (*iv*) the full structure. **b**-**e** Reflectance spectra of the four cases for TE- and TM-polarized light

To understand the role of the various optical components in the green scales, we performed finite-difference time-domain simulations on various, idealized photonic structures with varying grades of complexity (Fig. 8a). Sample parameters were extracted from electron micrographs, yielding a chitin layer thickness of *d*_c_ = 150 nm with an air gap of *d*_a_ = 80 nm. The thickness and spacing of the melanized ridges was 200 nm and 800 nm, respectively. The regular spaced air holes (also with a mean spacing of 800 nm) were estimated to have a diameter of 105 nm and a lateral spacing of 230 nm (i.e., *d*_c_ + *d*_a_).

Normal illumination of an ideal chitin-air multilayer structure (Fig. 8a, *i*) causes a polarization-independent reflectance spectrum with a maximum of ~0.99 at 630 nm (i.e., peak wavelength *λ*_max_ = 2 (*d*_c_*n*_c_ + *d*_a_) with a chitin refractive index *n*_c_ ≈ 1.55 [25]). Regular-spaced, orthogonal air slabs in the chitin-air multilayer (Fig. 8a, *ii*) invoke a minor form birefringence, but still a saturating reflectance at ~620 nm, with small oscillations in the reflectance spectrum (Fig. 8c). Putting melanin-containing baffles in the chitin-air multilayer (Fig. 8a, *iii*) has a severe optical effect, however, causing a pronounced polarization-dependency. The TE-reflectance spectrum shows a distinct peak in the green, at ~540 nm, with sidelobes at larger wavelengths, whereas the multi-lobed TM-reflectance spectrum peaks in the red, at ~720 nm (Fig. 8d). The multilayer with both the regular spaced air holes and the melanized baffles (Fig. 8a, *iv*) is a photonic structure as that found in the butterfly scales (Fig. 5). The spectra obtained by FDTD modelling (Fig. 8e) are indeed very similar to those measured experimentally (Fig. 6c). The TE-polarized reflectance is maximal in the green, at ~530 nm, with minor sidelobes in the yellow-red wavelength range, whereas the TM-polarized reflectance spectrum has multiple peaks with very similar reflectance values of ~0.3 at 520, 610 and 700 nm, respectively. The angle-dependent scattering calculated for this optical structure (Fig. 7g, h) corresponds closely to the experimentally observed scattering, showing strong grating-like diffraction (Fig. 7e, f).

## Discussion

The coloration toolkit of the male Rajah Brooke’s Birdwing, *Trogonoptera brookiana*, relies on a diversity of nanostructures, which, in combination with various absorbing pigments, create the strikingly vivid coloration of the butterfly. The optics of the pigmentary colored scales can be well understood as a basic combination of a thin film reflector and a strongly absorbing pigment. Papiliochrome pigment and multilayers determine the reflection properties of the red hairs; complex structured ridge multilayers and melanin-containing, absorbing baffles play a central role in the optics of the blue and green wing scales.

### Thin film reflectors in black and white scales

It is commonly assumed that the color of pigmented butterfly wing scales is due to the scattering of light by the wing scale’s irregular structures and that the wavelength-dependent absorbing pigment determines the scale’s color, because it acts as a high-pass spectral filter on the scattered light. We found that the white and black wing scales of *T. brookiana* have widely open windows and irregular cross-ribs (Fig. 2c, d). The lower lamina, acting as a thin film reflector, determines the reflectance spectrum of the white scales. The peak reflectance is at ~450 nm, indicating a lower lamina thickness of ~200 nm (Fig. 2e). This falls well in line with recent observations across all major butterfly families of the presence of thin film reflectors in pigmented wing scales [4, 8, 10, 11, 13]. For instance, the blue eye spots in the wings of the Peacock butterfly, *Inachis io* (Nymphalidae), were found to be created by pigmentless cover scales overlapping black ground scales, where the cover scales’ lower lamina acts as a blue-reflecting thin film. On the other hand, wing areas of the Peacock and other nymphalines where both cover and ground scales are pigmentless have a distinctly white color as a result from cumulative reflections of cover and ground scales as well as the wing substrate. The reflectance spectra of Fig. 1c and Fig. 2f are very similar to those measured in the blue and white wing areas of the nymphalines as well as in another papilionid, *Papilio xuthus* [26]. Yet, whereas the lower lamina of the blue scales of the nymphaline butterflies was a single, chitinous layer, the lower lamina of the bluish scales of *P. xuthus* was a multilayer consisting of two membranes with an air gap in between the membranes. In all cases, the scale’s reflectance spectrum, which depends on the thin film thickness (~150–250 nm), appears to be well-tuned to the absorbance spectrum of the scale’s pigment [4, 8, 10, 13, 26]. We note here that the anatomy of the black scales of *T. brookiana* is almost identical to that of the white scales, and their lower lamina also acts as a blue thin film reflector. However, a high concentration of melanin severely reduces the reflectance, thus causing the scales’ blackness.

### Complex photonic structures in *Trogonoptera*: ordered *Morpho*-type photonic structures

The photonics of the blue and green wing scales is remarkably complex and seems to have uniquely evolved in the animal kingdom. Compared to the well-known *Morpho* butterflies, two main differences are obvious: the order of the ridges is extremely high, and baffles of melanin within the ridges affect the optical characteristics (Figs. 4 and 5). *Morpho* butterflies show extreme blue iridescence due to a disordered multilayer, with severe height and position disorder of the ridge layers [36–38], causing the iconic Christmas tree structure of transmission electron micrographs [12]. Light diffraction by the ridges causes the line-like pattern in scatterograms [34].

What is the optical advantage of the extreme order in the green wing scales of *Trogonoptera brookiana*? First of all, the ordered ridges invoke form birefringence resulting in a strong polarization-dependent reflectance (Figs. 6, 7 and 8), which is much less in butterflies with similar, disordered architectures. Secondly, the order invokes a grating-like, spatially restricted reflection pattern, resulting in extreme color changes with minute changes in viewing angle (Fig. 6d, e). Similar optical effects have been noticed in other organisms, like birds-of-paradise, but the underlying structures have very different topologies [39, 40]. Thirdly, the presence of melanin in the ridges adds a novel optical mechanism that severely changes the spectral response and shapes the spatial reflections.

The rather extreme scale curvature of the green scales, with their very convex tip, spreads the light reflected by the grating structure into the hemisphere along the long axis of the scale (Fig. 7a-d). Scale curvature is not unusual in structured butterfly wing scales and is usually attributed to maximizing the viewing angle and/or shifting and spreading the viewing direction, as was demonstrated in some pierid [32] or riodinid [41] butterfly species having photonic structures similar to those encountered in *Trogonoptera brookiana*.

### Biological significance

The coloration of Rajah Brooke’s birdwings appears to be developed in different ways. As noted above, each scale type is optimized for a specific signaling purpose using various photonic structures. The prominent tooth-shaped green markings that are contrasted by the jet-black wing borders will likely serve as a potential aposematic signal to predators, similar to other strongly colored markings of related papilionids [2, 7, 42–44]. Especially so, since larval stages of *T. brookiana* have poisonous Aristolochiaceae as their main foodplants [1]. Furthermore, the *Trogonoptera brookiana* phenotype is unique amongst the birdwing butterflies and the striking pattern is probably associated to its unique communal behaviour where males often assemble in large groups [45].

Whether or not the wing scale colors are tuned by evolutionary selection for intraspecific recognition is a challenging question. Sexual dichromatism is present in birdwing butterflies where females generally possess a smaller number of color markings of smaller wing area compared to the males. In *Trogonoptera brookiana* the sexual dichromatism is far less distinctive than that of other sympatric birdwing species (e.g., *Troides* or *Ornithoptera* [7]). Female *T. brookiana* carry green wing patches on the dorsal wings, quite similar to the male, however in females these green areas are associated with an extensive white pattern. Furthermore, the discodial area of the hindwing is completely blue in the hindwing, but in the male these blue lines are restricted to the basal areas of the wing. When in motion, these different patterns will most likely emit quite a different signal.

Sexual dichromatism functions in mate recognition, enabled by a rich set of spectral photoreceptors [46]. Most likely *Troides* and *Trogonoptera*, which are in the same tribe (Troidini) in the family of Papilionidae [1, 2], evolved quite similar sets of spectral receptors. For the Golden Birdwing butterfly, *Troides aeacus formosanus*, Chen et al. [47] determined by intracellular recordings seven different photoreceptor types, with spectral sensitivities ranging from the UV to red. A comparison of the reflectance spectra of the colored wing areas in *T. brookiana* with the spectral sensitivities of the different photoreceptors indicates a stark spectral contrast of the various wing areas with the surrounding black framing of melanized scales (Fig. 1a, b). This indeed suggests that the wing colors are tuned to the butterfly’s visual system; similar to what has been previously observed in related butterflies [7].

### Wing scale development

The degree of order in the green and blue scales, but also the multilayering of the red bristles, is quite remarkable. All butterfly wing scales are hypothesized to develop via a similar pathway by the (out-)folding of cell membranes and organelles (see Ghiradella’s seminal studies [9, 29, 48]) as well as by the preferential alignment of intracellular F-actin fiber networks [49]. For the folding of multilayered ridges, Ghiradella’s observations of developing wing scales indicate that the multilayers are formed by elastic buckling of the cell membrane and subsequent backfilling with nascent cuticle [9, 29, 48]. Drying of this chitinous cuticle after cell apoptosis results in the final, highly anisotropic cell shape [49], as those observed in the SEM images of Figs. 2, 3, 4 and 5. The deposition of the melanin in the ridge centers will most likely happen subsequent to the buckling process. It will be of extreme interest to further investigate which cellular parameters control or drive the cellular processes on the nanoscale given the unusual degree of order observed in these wing scales, especially because the order must be the result of evolutionary selection.

### Bio-inspired replicas

Recently extensive nanotechnological attempts have been undertaken to mimic the photonic structures of butterfly scales in materials with novel optical effects and/or advanced functionalities. The exact fabrication of complex three-dimensional topologies is still beyond current nanofabrication capabilities, however [50–52]. The wing scales of *T. brookiana* have served as templates for creating light trapping structures based on the black wing scales via an inverse SiO_2_ replica [21, 23, 24]. Although improperly described at some points, the latter authors show that replication of the black scales results in black optical structures with similar morphology. The green wing scales could inspire the production of highly efficient gas sensors, due to the large surface area, similar to the sensors based on the well-structured, multilayered wing scales of *Morpho* butterflies [53–55].

## Conclusions

In conclusion, the coloration mechanisms of Rajah Brooke’s Birdwing consist of thin films, multilayers and higher dimensional photonic structures that are extremely ordered at the nanoscale, resulting in the birdwing’s stunning coloration. We for the first time observed structural color in butterfly hairs and observed that the wing scales of the showy green patches provide yet another example of uniquely arranged structures that create reflection patterns with strong polarization contrast, thus expanding our insight into biophotonic coloration, especially in insects. The birdwings’ novel, nanoscale-controlled structure may well provide inspiration for biomimetic applications.

## Methods

### Specimen

The investigated specimens of Rajah Brooke’s Birdwing, *Trogonoptera brookiana albescens* (Rothschild, 1895) (Lepidoptera: Papilionidae: Troidini), were obtained from Worldwide Butterflies (Dorset, UK; www.wwb.co.uk).

### Spectroscopy

Reflectance spectra of the wings of intact butterflies were measured (in air) with a bifurcated fiber-optic probe. The probe comprised six light guides, delivering light from a halogen-deuterium source (AvaLight-D(H)-S-bal; Avantes, Eerbeek, the Netherlands), which surrounded a central fiber that acts as a light collector of reflected light (of a spot with diameter ~1 mm) and which delivered it to a fiber optic spectrometer (Maya2000Pro; Ocean Optics, Duiven, the Netherlands). A white diffusing reflectance standard (Ocean Optics WS-1) served as the reference.

Absorbance spectra of single wing scales immersed in refractive index matching fluid as well as polarization-dependent reflectance spectra of single scales were measured with a custom-built microspectrophotometer. The light beam of a xenon light source was coupled with a quartz lens into the microscope, equipped with an Olympus 20x/0.45 objective. The spectral range of the microspectrophotometer was limited to wavelengths ≥ 360 nm.

### Scanning electron microscopy

The ultrastructure of the wing scales was investigated with a Tescan MIRA 3 LMH field-emission scanning electron microscope (Tescan, Brno, Czech Republic). To prevent charging, the samples were sputtered with a thin layer of palladium or gold prior to imaging.

### Transmission electron microscopy

For transmission electron microscopy (TEM) of the scales, wing parts were prefixed in 2 % paraformaldehyde and 2.5 % glutaraldehyde in 0.1 mol l^−1^ sodium cacodylate buffer (CB, pH 7.3) for ∼ 45 min. After dehydrating with a graded series of ethanol and infiltration with propylene oxide, the tissues were embedded in Spurr's resin. The tissues were cut into 50 nm ultrathin sections, double-stained with uranyl acetate and lead citrated and observed using a Hitachi H7650 (Tokyo, Japan) transmission electron microscope (as outlined in [34]).

### Imaging scatterometry

The hemispherical far-field light scattering pattern of single scales was visualized with an imaging scatterometer [14, 34, 56]. The scatterometer is built around an ellipsoidal mirror, which collects light from a full hemisphere around its first focal point, where the sample is positioned. Illumination was with a white light source (a xenon lamp), which delivered a narrow aperture (~5°) beam. For polarisation-dependent measurements, a linear sheet polarizer was added to the light path of the secondary beam. A small piece of magnesium oxide served as a white diffuse reference object. Images were acquired with an Olympus DP-70 camera and were subsequently corrected for geometrical distortions using a MATLAB routine.

### Finite-difference time-domain simulations

The polarization-dependent light scattering of various models was simulated with three-dimensional finite-difference time-domain (FDTD) calculations. We used Lumerical FDTD Solutions 8.15, a commercial-grade Maxwell equation solver. Each model was placed in a three-dimensional simulation volume of 2x10x6 μm^3^. The light source covered a wavelength range of 350 to 800 nm. For the refractive index dispersion of melanin and chitin we used previously published data [25, 57].
